# Integrated Transcriptome and Metabolome Analyses Reveal Complex Oxidative Damage Mechanisms in Rice Seedling Roots Under Different Carbonate Stresses

**DOI:** 10.3390/antiox14060658

**Published:** 2025-05-30

**Authors:** Yang Cao, Fei Hao, Jingpeng Li, Bolun Zhang, Zeming Li, Tiantian Liu, Yan Gao, Xuguang Niu, Xiaohu Liu, Hui Zhang, Lijuan Yang

**Affiliations:** 1 College of Land and Environment, Shenyang Agricultural University, Shenyang 110866, China; caoyang@stu.syau.edu.cn (Y.C.); haofei@stu.syau.edu.cn (F.H.); zhangbolun@stu.syau.edu.cn (B.Z.); 2022240565@stu.syau.edu.cn (Z.L.); 2023240601@stu.syau.edu.cn (T.L.); gaoyan2915@163.com (Y.G.); xuguangniu@syau.edu.cn (X.N.); liuxiaohu-mail@163.com (X.L.); 2 Northeast Institute of Geography and Agroecology, Chinese Academy of Sciences, Changchun 130102, China; lijingpeng@iga.ac.cn; 3 Da’an Sodic Land Experiment Station, Chinese Academy of Sciences, Da’an 131302, China; 4 National Engineering Research Center for Efficient Utilization of Soil and Fertilizer Resources, Shenyang 110866, China

**Keywords:** rice (*Oryza sativa* L.), carbonate stresses, seedling growth, reactive oxygen species, transcriptome, metabolite, antioxidants

## Abstract

Alkaline stress (AS) is one of the major threats that severely affects rice growth and grain yield. However, the differences in the damage caused by the main components of soda saline-alkali land, sodium carbonate (Na_2_CO_3_), and sodium bicarbonate (NaHCO_3_) to rice seedlings are still unclear. This study explored the effects of different carbonate stresses (Na_2_CO_3_ and NaHCO_3_) on rice seedling growth, root damage, physiological responses, and molecular changes. By administering equivalent concentrations of sodium ions through these different carbonate treatments, we observed that both stresses significantly inhibited rice growth. However, the inhibitory effect was more pronounced under the Na_2_CO_3_ treatment. Compared with the NaHCO_3_ treatment, Na_2_CO_3_ stress caused more severe damage to root cell membranes and led to a substantial decline in root vigor. Moreover, the contents of reactive oxygen species (ROS) and malondialdehyde (MDA) were markedly increased, indicating that Na_2_CO_3_ induces more severe oxidative damage. Transcriptomic and metabolomic analyses revealed a greater number of differentially expressed genes (DEGs) and differentially expressed metabolites (DEMs) in the Na_2_CO_3_ treatment group. The integrative analysis and validation demonstrated that pathways related to auxin, ascorbate, flavonoids, and glutathione metabolism were particularly enriched under Na_2_CO_3_ stress. These findings suggest that Na_2_CO_3_ stress may interfere with auxin signaling pathways and exerts a more profound impact on endogenous antioxidant systems, affecting rice growth at multiple levels. In summary, this research highlights the differential impacts of Na_2_CO_3_ and Na_2_CO_3_ stresses on rice seedling growth, physiology, and molecular processes, particularly oxidative damage and antioxidant responses. The insights gained provide a valuable theoretical foundation for enhancing rice alkali tolerance and developing strategies for the rational cultivation of rice in saline-alkaline soils.

## 1. Introduction

Soil salinization and alkalization have garnered widespread attention in the global scientific community, particularly in the fields of soil science, plant nutrition, and environmental science. The increasingly severe problem of salinized and alkalized soils not only presents a worldwide ecological challenge but also significantly hampers agricultural production [1,2]. The total area of saline and alkaline land globally is approximately 954 million hectares, while China’s saline and alkaline land covers 99.13 million hectares [3,4], accounting for about 10% of the country’s total land area. In Northeast China, one of the main types of saline-alkaline land is soda-saline land, which exceeds 3.42 million hectares in the region [5]. Soda saline-alkali land is primarily composed of high concentrations of carbonate, including Na_2_CO_3_ and NaHCO_3_ [6,7]. In addition to inducing osmotic stress and ion toxicity similar to those caused by neutral salt stress, carbonates also cause high pH injury, with the pH ranging from 8.5 to 11 [8]. Therefore, compared to neutral salts such as NaCl and Na_2_SO_4_, carbonate stress is more detrimental to plants.

The soils of soda-saline land, due to the presence of sodium bicarbonate and sodium carbonate, exhibit elevated pH levels. High pH can damage root tissue, impede the normal physiological functions of root cells, and affect the mineralization of organic materials (such as carbon, nitrogen, and phosphorus), which reduces nutrient cycling and supply, thereby inhibiting plant growth [9,10,11,12]. Salt stress inhibits plant growth and induces the accumulation of reactive oxygen species (ROS), malondialdehyde (MDA), etc.; in contrast, alkaline stress causes more severe inhibition of plant growth and results in greater damage [13,14,15]. This indicates that high pH is a major factor that contributes to the greater damage caused by alkali stress compared with saline stress [13,14]. Under salt stress and alkali stress, plants experience an increase in reactive oxygen species (ROS), which leads to oxidative stress, resulting in the degradation of lipids, proteins, and nucleic acids, adversely affecting the organelles and tissues of stems and roots [6,16,17]. However, the ROS production induced by alkaline stress is higher, and the interference with ROS scavenging systems is greater than that induced by salt stress [6,13,18]. Studies on rice have shown that alkaline stress can cause serious damage to roots, leading to cell death and plant wilting, ultimately resulting in plant death due to the imposed high pH stress [6,13,19]. Research has found that applying exogenous antioxidants or plant hormones such as abscisic acid (ABA) can enhance the antioxidant capacity of plants, reduce ROS accumulation, and alleviate alkali stress damage [6,16,20], indicating that root damage and cell death are primarily due to the accumulation of ROS in roots triggered by AS.

Plant cells can neutralize ROS through a complex antioxidant defense system, which includes both enzymatic and non-enzymatic components [15,21]. Enzymatic antioxidants include superoxide dismutase (SOD), catalase (CAT), and peroxidase (POD), while non-enzymatic antioxidants include ascorbic acid (AsA), glutathione (GSH), flavonoids, alkaloids, and phenolic acids. These components play crucial roles in protecting plants from oxidative damage caused by abiotic stresses, such as SS and AS [22,23]. SOD acts as the primary defense mechanism by converting O_2_^−^ into H_2_O_2_. H_2_O_2_ is subsequently detoxified to water (H_2_O), either through CAT, APX, or by participating in the AsA-GSH cycle. Flavonoids and alkaloids directly scavenge ROS [23,24]. The balance between ROS production and elimination can be severely disrupted by various stressors, leading to excessive accumulation of intracellular ROS and subsequent damage to plant cells [6,20]. In highly alkaline conditions, the severe inhibition of plant growth has been linked to impaired antioxidant systems [1]. Therefore, maintaining ROS homeostasis mediated by the antioxidant system is a critical factor for plants to adapt to alkaline and salt stresses.

Carbonates are the main components causing soil alkalization, with sodium carbonate (Na_2_CO_3_) and sodium bicarbonate (NaHCO_3_) being the two most common forms. Now, many researchers have commenced studies to elucidate the damage caused by alkali stress to plants and the mechanisms of plant tolerance to alkalinity [25,26]. These studies have mainly focused on treatments with sodium bicarbonate [27,28] or sodium carbonate to simulate high pH [29,30]; mixed treatments of sodium bicarbonate and sodium carbonate to mimic soda-saline soils [9]; and comparisons with neutral salt treatment involving sodium chloride [14,21]. Rice (*Oryza sativa* L.) cultivation is a highly effective biological method for reclaiming saline-sodic soils [31]. Planting rice not only removes salts from the soil through irrigation but also significantly reduces the negative impact of soil structure deterioration [5]. However, there remains ambiguity concerning the differential damage to rice caused by sodium bicarbonate, a weakly alkaline salt, versus sodium carbonate, a strongly alkaline salt.

This study aims to systematically evaluate the effects of sodium carbonate and sodium bicarbonate on the growth of rice seedlings and root damage, while also analyzing their impacts on ROS accumulation and the antioxidant system. Furthermore, we will uncover the molecular mechanisms of rice under different carbonate stress conditions through transcriptomic and metabolomic analyses. These findings will provide valuable scientific insights into enhancing the alkalinity resistance of rice through antioxidant mechanisms.

## 2. Materials and Methods

### 2.1. Plant Materials and Experimental Design

The study utilized an alkali-tolerant rice variety, Dongdao4, provided by the Northeast Institute of Geography and Agroecology, Chinese Academy of Sciences. The rice seeds were disinfected by soaking them in 75% (*v*/*v*) ethanol for 5 min, followed by thorough rinsing with distilled water. Subsequently, the seeds were transferred to a breathable tissue culture bottle filled with distilled water and incubated at a constant temperature of 28 °C for 48 h. After soaking, the excessive distilled water was drained, and the seeds were placed in a plastic Petri dish to maintain moisture and allowed to germinate in the dark at 30 °C for 24 h.

Seeds exhibiting uniform germination were grouped into sets of 18 seeds, which were sown in three rows within PCR tubes, with the tube bottoms cut off. These PCR tubes were placed in disposable plastic cups (320 mL capacity) filled with distilled water for a 7-day culture period, followed by another 7 days of growth with a 1/2 Kimura nutrient solution. The alkali stress treatment was initiated after a 2-week pre-culture period. The growth conditions in the artificial climate chamber were set to a light–dark cycle of 12 h each, with temperatures of 25 °C during the light phase and 20 °C during the dark phase, as well as a light intensity of 350 µmol·m^−2^·s^−1^.

Different concentrations of Na_2_CO_3_ and NaHCO_3_ alkaline solutions were selected in this experiment to simulate the effects of alkaline soil on rice seedlings. The concentration of Na_2_CO_3_ was selected based on the experimental results of Zhang et al. and Lv et al. [6,13], while the concentration of NaHCO_3_ was selected based on the concentration of Na_2_CO_3_ under the condition of equal sodium ion concentration. The pH of the alkaline stress solution was measured using a pH700 acidimeter, and the EC was measured using a DDS-307A conductivity meter.

The following five treatment groups were established for this experiment: (1) control with distilled water (CK); (2) treatment with 5 mmol/L Na_2_CO_3_ (5Na, pH 11.18; EC 1.04 mS/cm); (3) treatment with 15 mmol/L Na_2_CO_3_ (15Na, pH 11.39; EC 2.72 mS/cm); (4) treatment with 10 mmol/L NaHCO_3_ (10NaH, pH 8.52; EC 0.86 mS/cm); and (5) treatment with 30 mmol/L NaHCO_3_ (30NaH, pH 8.68; EC 2.58 mS/cm). Each treatment was replicated three times. On the 1st, 3rd, and 5th day of alkali stress, 0.1 g of fresh rice root was harvested from each treatment, cut into 0.3 cm segments using sterile scissors, rapidly frozen in liquid nitrogen, and stored at −80 °C for the subsequent RNA extraction, RNA sequencing, and metabolomic, physiological, and biochemical index analyses.

### 2.2. Measurement of Rice Seedling Growth Index

After 5 days of alkali stress treatment, ten rice seedlings were randomly selected from each treatment group, and their average height and fresh weight (FW) were recorded. Each treatment was replicated three times, involving a total of 30 seedlings.

### 2.3. Assessment of Root Physiological Indices in Rice Seedlings

#### 2.3.1. Plasma Membrane Integrity (MI) and Root Vigor Measurement

To evaluate MI, rice root segments were placed in 50 mL centrifuge tubes and submerged in deionized water for 2 h at room temperature. The initial conductivity (C1) was measured using a conductivity meter (DDS-307A, Shanghai, China). The centrifuge tube was then subjected to boiling for 40 min, cooled, and the final conductivity (C2) was measured. The MI (%) was calculated using the following formula: MI (%) = (C1/C2) × 100% [32].

Root vigor was assessed using the 2,3,5-triphenyltetrazolium chloride (TTC) reduction method. The reduction of TTC to form red insoluble triphenylmethyl bromide was measured by reading the OD value at 485 nm with a spectrophotometer (UVmini-1240, Shimadzu, Japan) [32].

#### 2.3.2. Measurement of Reactive Oxygen Species (ROS) and Lipid Peroxidation

The content of superoxide anions (O_2_^−^) was determined by the hydroxylamine oxidation method. The reaction between superoxide and hydroxylamine produced NO_2_^−^, which subsequently reacted with *p*-aminobenzenesulfonic acid and α-naphthylamine to form a red product, measurable at 530 nm [33]. The H_2_O_2_ content was assessed by the formation of a yellow titanium peroxide complex, with the OD measured at 415 nm [34]. The malondialdehyde (MDA) content was evaluated using the thiobarbituric acid method, with the absorbance determined at 532 nm and 600 nm [35].

#### 2.3.3. Antioxidant Enzyme Activity Determination

Fresh rice roots (0.1 g) were homogenized in a 50 mM phosphate buffer (pH 7.8) to prepare a crude enzyme solution, which was centrifuged at 12,000 rpm for 15 min at 4 °C. The activities of superoxide dismutase (SOD), peroxidase (POD), and catalase (CAT) were analyzed using established methods and spectrophotometer readings at the following specified wavelengths: 560 nm for SOD, 470 nm for POD, and 240 nm for CAT [36]. Glutathione reductase (GR) activity was assessed based on the decrease in NADPH absorbance at 340 nm using a glutathione reductase (GR; Michy Biomedical, M0303A, Suzhou, China) assay [37].

#### 2.3.4. Measurement of Plant Auxin (IAA) Content

For the IAA content measurement, enzyme-linked immunosorbent assay (ELISA) techniques were employed, with the absorbance read at 450 nm using an ELISA kit specific to plant growth hormones (IAA; JingMei Biotechnology, JM-01121P2, Jiangsu, China).

#### 2.3.5. Measurement of Reduced Ascorbic Acid (AsA) Content

The content of reduced ascorbic acid was evaluated based on its ability to reduce ferric ions (Fe^3+^) to ferrous ions (Fe^2+^), forming a red chelate measurable at 534 nm [38] using a reduced ascorbic acid (AsA; Michy Biomedical, M0401A, Suzhou, China) kit.

#### 2.3.6. Measurement of Glutathione (GSH and GSSG) Contents

The GSH content was determined using a DTNB-based assay, measuring the absorbance at 412 nm [39] using a reduced glutathione (GSH; Michy Biomedical, M0301A, Suzhou, China) kit. For GSSG, the interference from the GSH was eliminated, and the GSSG content was calculated based on the GR-catalyzed reactions monitored at 412 nm using an oxidized glutathione (GSSG; Michy Biomedical, M0302A, Suzhou, China) kit.

#### 2.3.7. Measurement of Flavonoid Content

The flavonoid content was determined by the formation of a red complex with aluminum ions in alkaline nitrite solution, with the absorbance measured at 510 nm [40] using a plant flavonoid (Flavonoid; Michy Biomedical, M0118A, Suzhou, China) kit.

### 2.4. Transcriptomic Analysis

For the transcriptomic analysis, 0.2 g of rice roots from the CK, 5Na, and 10NaH treatments was collected in biological triplicates. The total RNA was extracted using TRIzol reagent and treated with DNase I. The RNA quality was assessed using an Agilent 2100 Bioanalyzer (Rocklin, CA, USA) and a NanoDrop spectrophotometer (Wilmington, USA). The cDNA library was sequenced using the Illumina Novaseq 6000 platform at Allwegene Biotechnology (Beijing, China). Clean readings were mapped to the rice reference genome using TOPHAT, and differentially expressed genes (DEGs) were identified with DEGseq2. Gene Ontology (GO) and Kyoto Encyclopedia of Genes and Genomes (KEGG) analyses on DEGs were conducted using online tools.

### 2.5. Metabolomics Analysis

The metabolomics analysis involved extracting 0.2 g of rice roots from the CK, 5Na, and 10NaH treatments, with six biological replicates per group. Samples were prepared and analyzed in accordance with standard protocols, extracting metabolites using a 50% methanol solution. The extracts were analyzed using ultra-high-performance liquid chromatography (UPLC) coupled with mass spectrometry, utilizing an ACQUITY-UPLC-T3 column. Differentially expressed metabolites (DAMs) were identified based on their VIP scores and *p*-values from *t*-tests.

### 2.6. RNA Extraction and Real-Time Quantitative PCR (qRT-PCR)

For the qRT-PCR, the total RNA was extracted from rice roots using TRIzol reagent (ProbeGene, MM032, Jiangsu, China), and first-strand cDNA was synthesized using a specific kit. Gene-specific primers were designed using Primer 5.0 software. The housekeeping gene β-actin was employed as an internal control, and the relative expression levels were calculated using the 2^−ΔΔCT^ method.

### 2.7. Statistical Analysis

Statistical analyses were performed using SPSS 27.0. One-way ANOVA and Duncan’s multiple range tests were utilized to determine significant differences, with *p*-values < 0.05 considered statistically significant. Data visualization was conducted using Origin2022, GraphPad Prism 8.0, and online analysis platforms.

## 3. Results

### 3.1. Effect of Different Carbonate Stresses on Rice Seedling Growth and Root Injury

**Figure 1:**
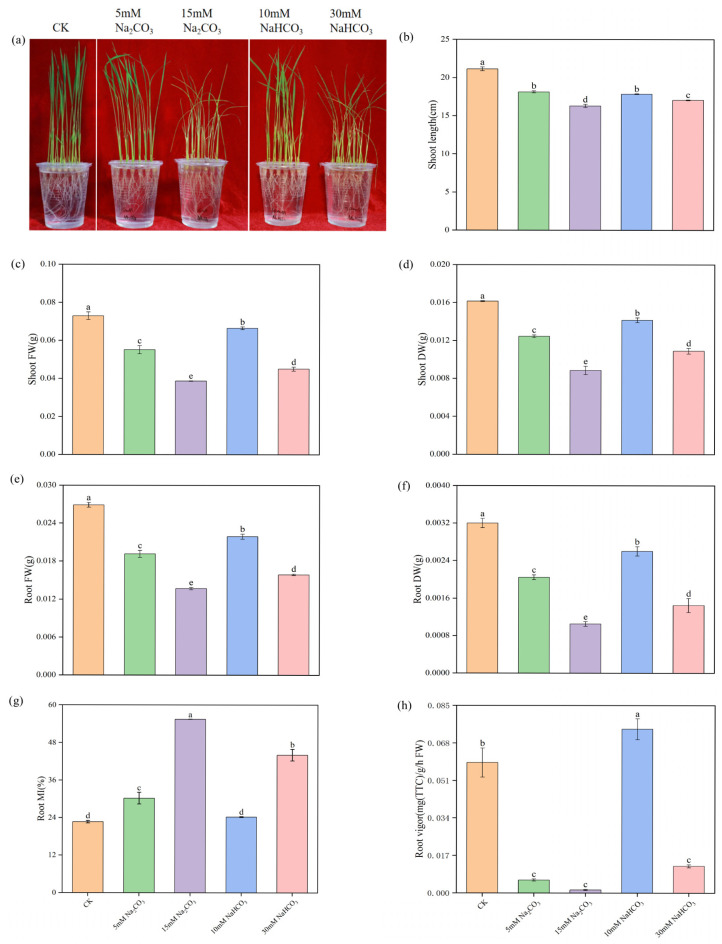
Effects of different carbonate stresses on the phenotype and physiology of rice seedlings: (**a**) phenotype; (**b**) shoot length; (**c**) shoot fresh weight (FW); (**d**) shoot dry weight (DW); (**e**) root fresh weight (FW); (**f**) root dry weight (DW); (**g**) root MI; (**h**) root vigor. Data are the mean ± SD of three biological replicates. Lowercase letters indicate a statistically significant difference (*p* < 0.05) by Duncan’s test.

To investigate the differences in damage to rice seedlings under various carbonate stresses, we first analyzed the effects of the Na_2_CO_3_ and NaHCO_3_ treatments on rice growth while maintaining an equal Na^+^ content. As shown in the growth photographs and shoot length measurements (Figure 1a,b), alkali stresses inhibited the growth of rice seedlings, with higher-concentration treatments exhibiting more severe inhibition. Moreover, the inhibition caused by the Na_2_CO_3_ treatments was greater than that caused by the NaHCO_3_ treatments. Compared to the control group, alkali stresses significantly reduced the biomass accumulation of the rice seedlings. The fresh and dry weights of both shoots and roots treated with low and high concentrations of Na_2_CO_3_ were significantly lower than those treated with NaHCO_3_ under equal Na^+^ ion conditions (Figure 1c–f). These results indicate that alkali stresses hinder the growth of rice seedlings, with the Na_2_CO_3_ treatments having a more pronounced inhibitory effect than the NaHCO_3_ treatments.

To assess the root damage caused by the alkali stresses, we analyzed the membrane injury (relative conductivity) and root vigor in the rice seedling roots. The alkali stresses significantly damaged the root cell membrane, increasing the electrolyte permeability of the plasma membrane (Figure 1g). Additionally, as shown in Figure 1h, the alkali stress significantly reduced root vigor compared with the control group, with the Na_2_CO_3_ treatments causing more severe inhibition of root vigor than the NaHCO_3_ treatments. Overall, these results demonstrate that the alkali stresses significantly inhibited seedling growth and injured the root system, with the Na_2_CO_3_ treatments causing greater damage than the NaHCO_3_ treatments.

### 3.2. Effects of Different Carbonate Stresses on Reactive Oxygen Species Accumulation and Antioxidant Enzyme Activity in Rice Roots

**Figure 2:**
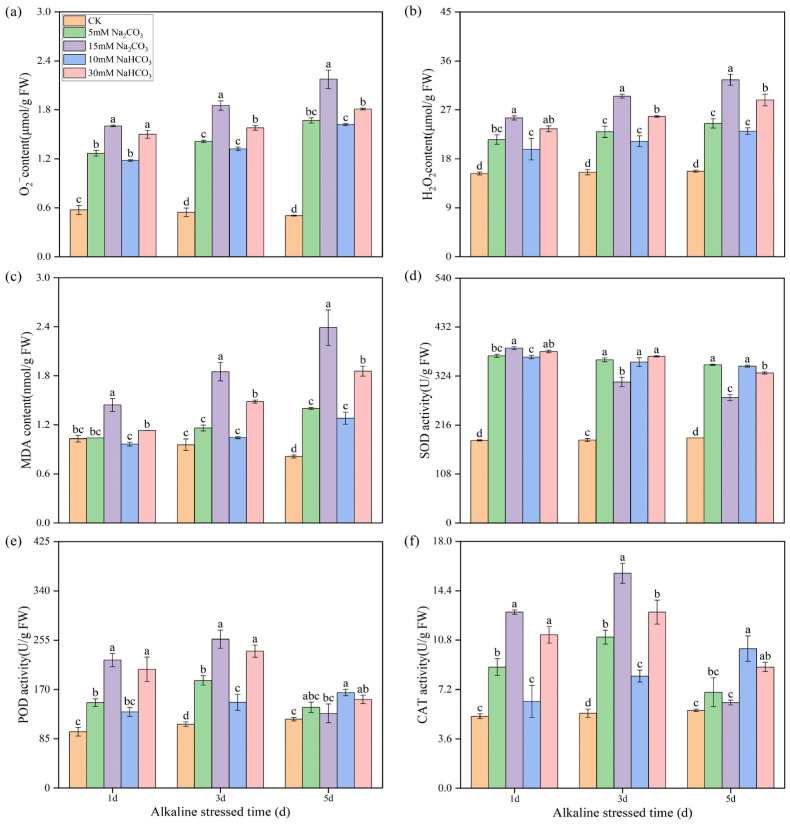
Effects of different carbonate stresses on reactive oxygen species accumulation and antioxidant enzyme activities in the roots of rice seedlings: (**a**) superoxide anion (O_2_^−^); (**b**) hydrogen peroxide (H_2_O_2_); (**c**) malondialdehyde (MDA); (**d**) superoxide dismutase (SOD); (**e**) peroxidase (POD); (**f**) catalase (CAT). Data are the mean ± SD of three biological replicates. Lowercase letters indicate a statistically significant difference (*p* < 0.05) by Duncan’s test.

To elucidate the differences in endogenous secondary oxidative stress, we measured the contents of reactive oxygen species (ROS) and malondialdehyde (MDA) in rice seedling roots following the various alkali stresses. Alkali stress significantly increased the accumulation of O_2_^−^ and H_2_O_2_ in rice roots, with the levels rising progressively over time. The accumulation of ROS induced by the high concentrations of Na_2_CO_3_ was significantly greater than that induced by the high concentrations of NaHCO_3_, while no significant differences were observed among the low-concentration alkali treatments (Figure 2a,b). The MDA content remained stable in the control groups but increased significantly in the alkali stress groups with prolonged treatment. Notably, the MDA contents in the high-concentration alkali stress groups were substantially higher than in the control groups, and the MDA contents in the low-concentration alkali stress groups were also elevated compared with the control group by the third day of treatment (Figure 2c). Interestingly, the ROS and MDA levels with the Na_2_CO_3_ treatments consistently exceeded those with the NaHCO_3_ treatments throughout the treatment period (Figure 2a–c). These findings indicate that the accumulation of ROS in rice root cells due to alkali stress leads to lipid peroxidation of cell membranes, with the Na_2_CO_3_ treatments resulting in more severe lipid peroxidation than the NaHCO_3_ treatments.

We also measured the activity of antioxidant enzymes. The superoxide dismutase (SOD) activity was significantly enhanced by alkali stress (Figure 2d). The SOD activity remained stable and high under low-concentration alkali stresses, while it decreased under high-concentration alkali stresses, particularly in the Na_2_CO_3_ treatments, where the SOD enzyme activity decreased most significantly. At day 1, the SOD activities in the high-concentration alkali treatments were notably higher than in the low-concentration treatments, but they significantly decreased by days 3 and 5. The activities of peroxidase (POD) and catalase (CAT) exhibited similar trends. The POD and CAT activities in the low-concentration NaHCO_3_ treatments gradually increased over time. In contrast, the POD and CAT activities in the sodium carbonate and high-concentration NaHCO_3_ treatments initially increased before declining from days 1 d to 5 d, with the activities in the high-concentration alkali treatments being distinctly higher than those in the low-concentration treatments (Figure 2e,f).

### 3.3. Transcriptome Profiling of Rice Seedling Roots Under Different Carbonate Stresses

**Figure 3:**
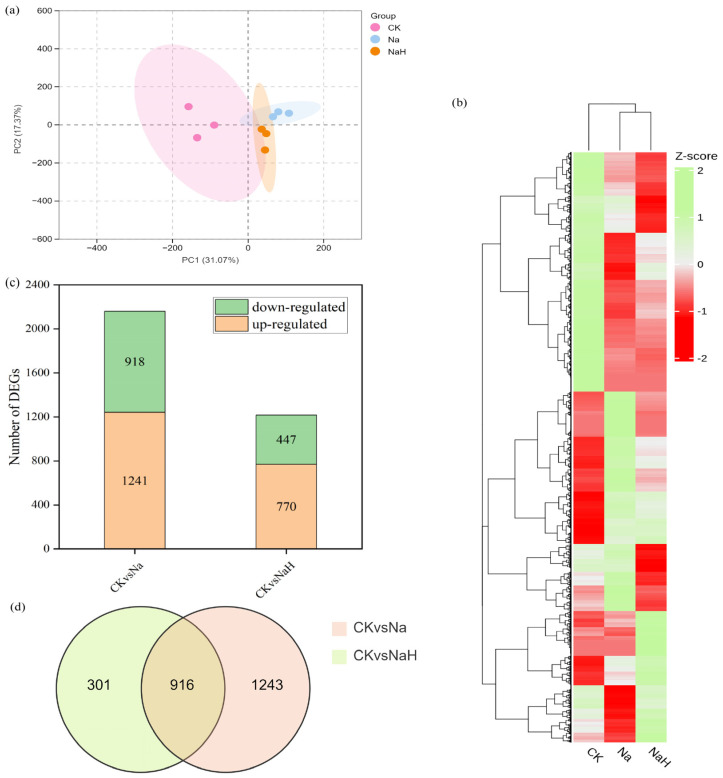
Transcriptomic data analysis of rice seedling roots under different carbonate treatments. (**a**) PCA of DEGs. CK group: pink dot; Na group: blue dot; NaH group: yellow dot. X−axis and Y−axis indicate the first and second principal components (PC1 and PC2), respectively. The score plots of PC1 and PC2 show cohesion within groups and the separateness of the CK, Na, and NaH groups. (**b**) Heatmap showing the results of the clustering analysis of the DEGs. (**c**) The number of DEGs. (**d**) Venn diagram of the DEGs.

To understand the effects of various carbonates on the transcriptome of rice seedling roots, we conducted a comprehensive transcriptome analysis. A total of 70.29 G of raw base data was generated, with 70.15 G of clean bases obtained after filtration. Each treated sample generated more than 7 G of clean base data, and the Q20 and Q30 quality metrics were both above 97% and 93%, respectively. The GC content ranged from 52% to 53.14%, indicating the high quality of our transcriptomic sequencing data (Table S1). Principal component analysis (PCA) and heat maps revealed significant differences among the control (CK), sodium carbonate (Na), and sodium bicarbonate (NaH) groups (Figure 3a,b).

To identify differentially expressed genes (DEGs) from the transcriptomic data, we utilized DESeq2 with the criteria of |log2 (fold change)| ≥ 1 and Padj < 0.05 for intergroup comparisons. This analysis yielded 2159 EGs in the CK vs. Na group, with 1241 genes upregulated and 918 genes downregulated. In the CK vs. NaH group, we identified 1217 DEGs, including 770 upregulated and 447 downregulated genes (Figure 3c). The Venn diagram analysis shows that the two comparison groups, CK vs. Na and CK vs. NaH, shared 916 overlapping DEGs under alkali stress (Figure 3d).

**Figure 4:**
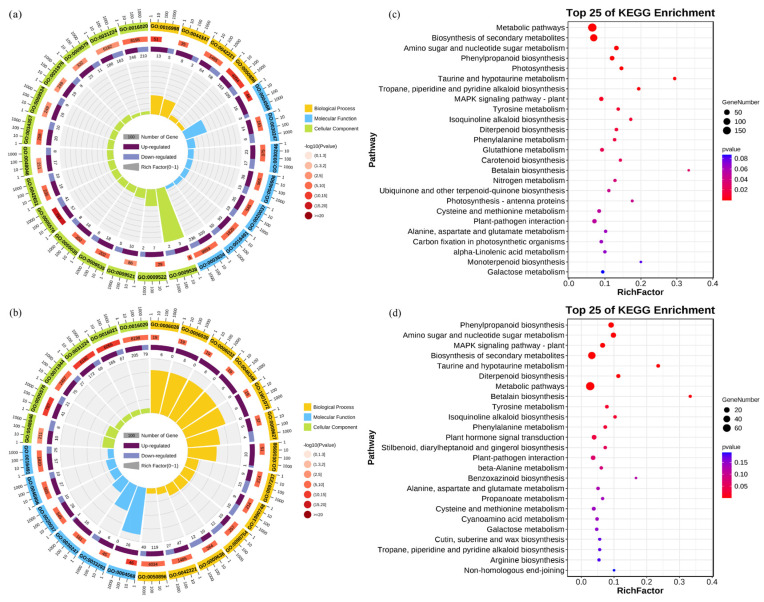
Enrichment analysis of DEGs. (**a**) GO enrichment analysis of the DEGs in the CK vs. Na group. (**b**) GO enrichment analysis of DEGs in the CK vs. NaH group. The first circle represents the enrichment classification, with the coordinates of the number of genes outside the circle. Different colors represent different categories, with yellow representing biological processes, blue representing molecular functions, and green representing cellular composition. The second circle represents the classification number and Q- or *p*-value of the background gene. The more genes there are, the longer the bar, the smaller the value, and the redder the color. The third circle is a bar chart of the proportion of genes, with dark purple indicating upregulation and light purple indicating downregulation. The specific values are displayed in the lower part. The fourth circle represents the Rich Factor values for each category, and each small grid in the background auxiliary line represents 0.1. (**c**) KEGG enrichment analysis of the DEGs in the CK vs. Na group. (**d**) KEGG enrichment analysis of the DEGs in the CK vs. NaH group. The *p*-value is presented as a color scale, the size of the dots represents the DEG number mapped for each pathway.

To elucidate the molecular mechanisms underlying the response of rice roots to alkali stress, we performed a Gene Ontology (GO) enrichment analysis and Kyoto Encyclopedia of Genes and Genomes (KEGG) analysis. The GO analysis revealed that the DEGs in the CK vs. Na group were primarily enriched in the biological processes related to responses to stimuli (GO: 0050896) and chemicals (GO: 0042221), as well as in molecular functions associated with oxidoreductase activity (GO: 0016491) and catalytic activity (GO: 0003824). In contrast, the DEGs in the CK vs. NaH group were mainly concentrated in the cellular components related to membranes, including the membrane (GO: 0016020), intrinsic membrane component (GO: 0031224), and integral membrane component (GO: 0016021) (Figure 4a,b, Table S2). Notably, the DEGs associated with responses to stimuli and chemicals were significantly higher in the CK vs. Na group compared to the CK vs. NaH group, indicating substantial differences in how rice seedling roots respond to sodium carbonate versus sodium bicarbonate stress. Furthermore, the KEGG analysis indicates that the DEGs in both the CK vs. Na and CK vs. NaH groups were predominantly involved in pathways related to secondary metabolite biosynthesis (ko01110), amino sugar and ribonucleotide metabolism (ko00520), and phenylpropanoid biosynthesis (ko00940) (Figure 4c,d, Table S3).

### 3.4. Metabolome Differences of Rice Seedling Roots Under Different Carbonate Stresses

**Figure 5:**
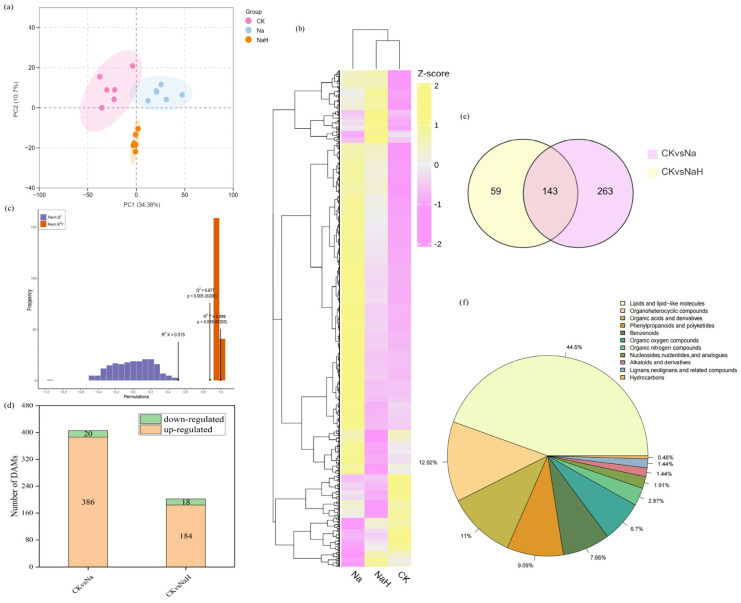
Metabolomics data analysis of rice seedlings roots under different carbonate stresses. (**a**) PCA of the DAMs. Pink dot, CK group; blue dot, Na group; yellow dot, NaH group. X−axis and Y−axis indicate the first and second principal components (PC1 and PC2), respectively. The score plots of PC1 and PC2 show cohesion within groups and separateness of the CK, Na, and NaH groups. (**b**) Heatmap showing the results of the clustering analysis of the DAMs. (**c**) The OPLS-DA model verification diagram of the DAMs. Q2 represents the prediction ability of the model, and R2Y represents the interpretation rate of the built model to the Y matrix. The closer the two indexes are to 1, the more stable and reliable the model. Q2 > 0.5 can be considered as an effective model, and *p* < 0.05 is the best model. (**d**) The number of DAMs. (**e**) Venn diagram of the DAMs. (**f**) Taxonomic map of metabolites.

To further investigate the effects of alkali stress on metabolites in rice roots, we conducted a non-targeted metabolomic analysis using UHPLC-MS/MS. Both the PCA and heat map analyses revealed significant differences in the metabolite profiles among the CK, Na, and NaH groups (Figure 5a,b). The orthogonal partial least squares discriminant analysis (OPLS-DA) further corroborated these findings (Figure 5c), indicating that alkali stress induces significant changes in the metabolite composition of rice roots. Based on the criteria of VIP > 1 and *p* < 0.05, we identified a total of 406 differently accumulated metabolites (DAMs) in the CK vs. Na group, of which 386 were upregulated and 20 were downregulated. In the CK vs. NaH group, we identified 202 differentially expressed metabolites, with 184 upregulated and 18 downregulated (Figure 5d). The Venn diagram analysis revealed that 143 DAMs were shared between the CK vs. Na and CK vs. NaH groups (Figure 5e). The detected metabolites were categorized as follows: lipids and lip-like molecules (44.50%); organoheterocyclic compounds (12.92%); organic acids and their derivatives (11.00%); phenylpropanoids and polyketides (9.09%); benzenoids (7.66%); organic oxygen compounds (6.70%); organic nitrogen compounds (2.87%); nucleosides, nucleotides, and analogues (1.91%); alkaloids and their derivatives (1.44%); lignans, neolignans, and related compounds (1.44%); and hydrocarbons (0.48%) (Figure 5f).

**Figure 6:**
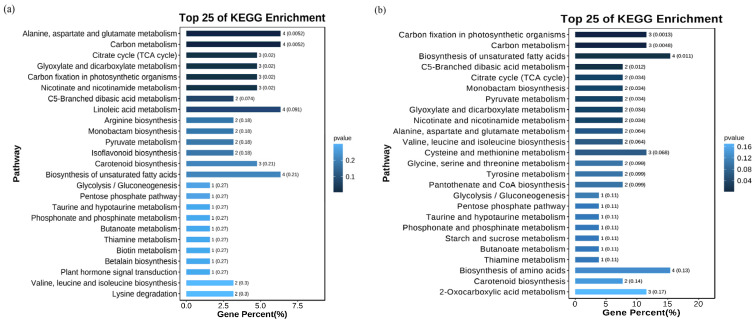
KEGG enrichment analysis of the DAMs: (**a**) KEGG enrichment analysis of the DAMs in the CK vs. Na group; (**b**) KEGG enrichment analysis of the DAMs in the CK vs. NaH group. The *p*-value is presented as a color scale, and the size of the dots represents the DAM number mapped for each pathway.

We utilized the KEGG database to annotate the differential metabolites (DAMs), and the results of the enrichment analysis revealed distinct patterns. In the comparison between the CK and Na groups, the DAMs were primarily enriched in the metabolic pathways related to alanine, aspartate, and glutamate metabolism; carbon metabolism; and linoleic acid metabolism (Figure 6a). Conversely, in the CK vs. NaH group, significant enrichment was observed in the pathways associated with carbon fixation, carbon metabolism, and the biosynthesis of unsaturated fatty acids in photosynthetic organisms (Figure 6b, Table S4). These findings highlight notable differences in the metabolic pathways of the DAMs between the Na_2_CO_3_ and NaHCO_3_ stresses.

### 3.5. Integrative Analysis of Transcriptome and Metabolome of Rice Seedling Roots Under Different Carbonate Stresses

**Figure 7:**
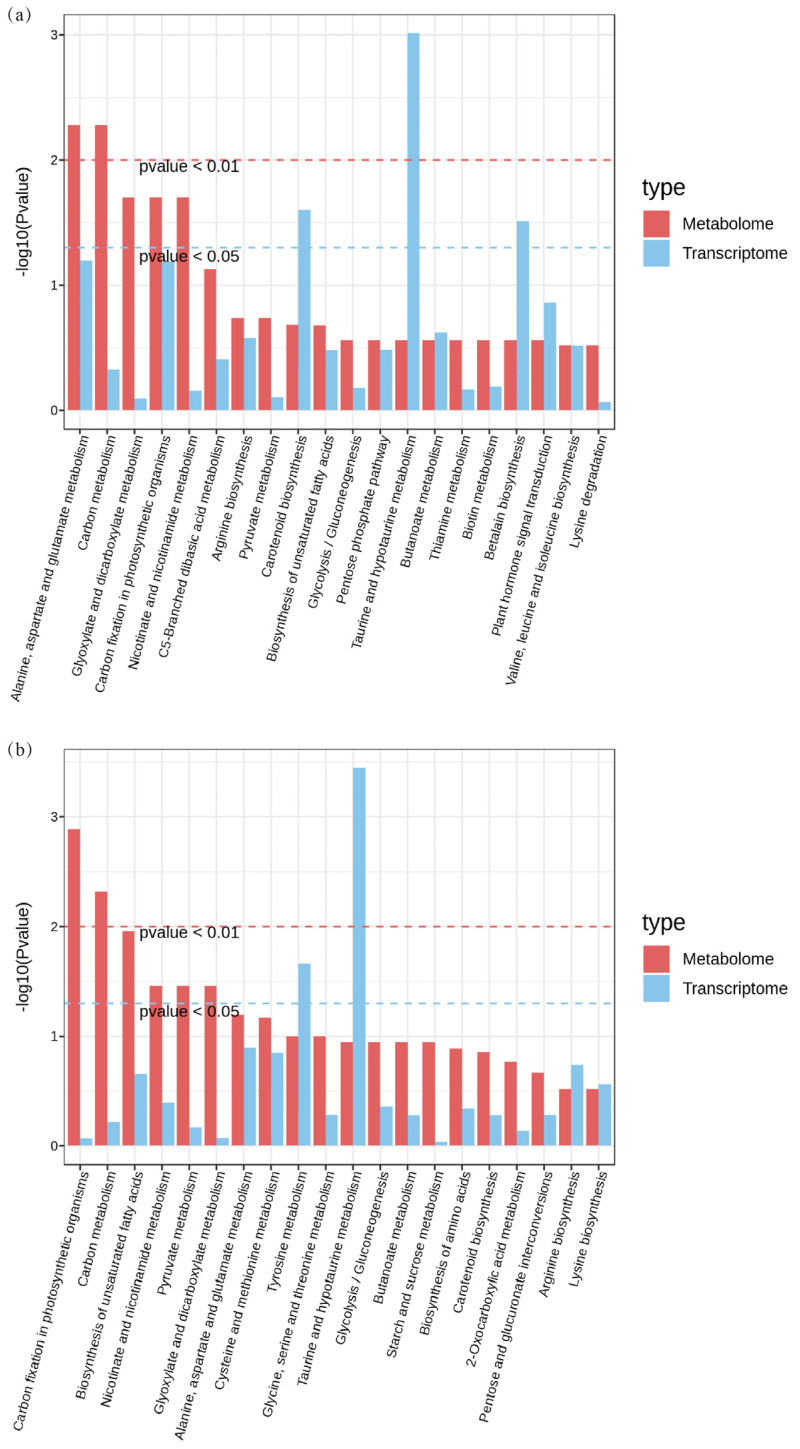
The top 20 prominent pathways for the integrative analysis of the transcriptome and metabolome of rice seedling roots under different carbonate stresses: (**a**) KEGG co-enrichment analysis of the DEGs and DAMs in the CK vs. Na formation; (**b**) KEGG co-enrichment analysis of the DEGs and DAMs in the CK vs. NaH formation. The red dotted line indicates that the *p* value < 0.01, and the blue dotted line indicates that the *p* value < 0.05.

To clarify the regulatory network under different carbonate stresses, we conducted integrated transcriptomic and metabolomic analyses. The KEGG enrichment analysis revealed 56 common enrichment pathways for the DEGs and DAMs in the CK vs. Na group and 33 enrichment pathways in the CK vs. NaH group (Table S5). The 20 most prominent pathways for each comparison group are illustrated in Figure 7. Commonly enriched pathways in both groups include the metabolism of alanine, aspartic acid, and glutamate, as well as carbon and glyoxylate metabolism.

**Figure 8:**
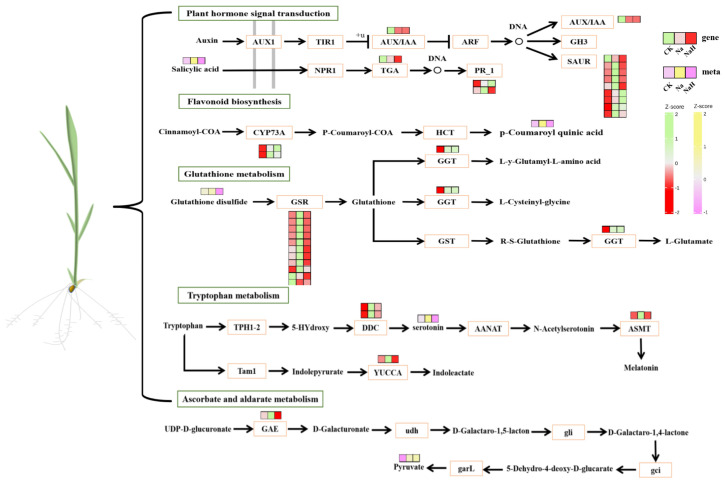
Changes in the main metabolic pathways in the roots of rice seedlings under sodium carbonate stress.

Furthermore, the DEGs and DAMs in the CK vs. Na group showed significant enrichment in pathways related to plant hormone (auxin) signal transduction, ascorbate and aldarate metabolism, glutathione metabolism, and flavonoid biosynthesis (Figure 8 and Table S6). Notably, these pathways were not enriched in the CK vs. NaH comparison group. These differences in pathway enrichment may contribute to the varying levels of stress damage observed between the Na_2_CO_3_ and NaHCO_3_ treatments.

### 3.6. Verification of DAMs and DEGs Related to Auxin, Ascorbate, and Flavonoid Biosynthesis and Glutathione Metabolism in Rice Seedling Roots Under Different Carbonate Stresses

#### 3.6.1. Effects of Different Carbonate Stresses on the Synthesis of Auxin and Antioxidants in Rice Seedling Roots

**Figure 9:**
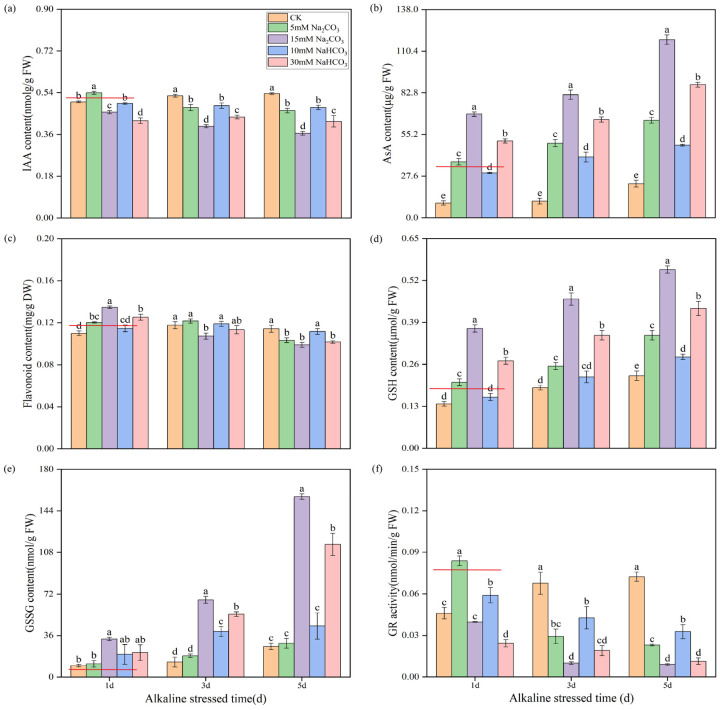
Verification DAMs related to auxin, ascorbate, flavonoid biosynthesis, and glutathione metabolism in rice seedling roots under different carbonate stresses: (**a**) auxin indoleacetic acid (IAA) content; (**b**) ascorbic acid (ASA) content; (**c**) glutathione reductase (GR) activity; (**d**) reduced glutathione (GSH) content; (**e**) oxidized glutathione (GSSG) content; (**f**) flavonoid content. Data are the mean ± SD of three biological replicates. Lowercase letters indicate a statistically significant difference (*p* < 0.05) by Duncan’s test. The red lines in the figure are used to express the differences among various treatments more intuitively.

To further validate the accuracy of our metabolomics data, we measured the contents of auxin, ascorbic acid, flavonoids, and glutathione and the activity of glutathione reductase (GR). Under alkali stress conditions, we observed that the synthesis of indole-3-acetic acid (IAA) was increasingly inhibited over time. Notably, rice seedling roots treated with a high concentration of Na_2_CO_3_ exhibited a significantly lower IAA content compared to that treated with a high concentration of NaHCO_3_ (Figure 9a). This suggests that Na_2_CO_3_ stress has a more pronounced inhibitory effect on auxin synthesis.

Additionally, alkali stress significantly increased the ascorbic acid content in the roots of rice seedlings, with the levels rising over time. Importantly, the ascorbic acid content was significantly higher with the Na_2_CO_3_ treatment compared to the NaHCO_3_ treatment under isosodium ion conditions (Figure 9b). In the initial stages of treatment, alkali stress led to an increase in flavonoid content; however, this content gradually decreased over time, with the reduction being more significant in the Na_2_CO_3_ treatment compared with the NaHCO_3_ treatment (Figure 9c).

Both reduced glutathione (GSH) and oxidized glutathione (GSSG) levels increased progressively over time under alkali stress, with the Na_2_CO_3_ treatment yielding higher concentrations than the NaHCO_3_ treatment (Figure 9d,e). The activity of glutathione reductase (GR) exhibited a significant decrease with prolonged exposure to Na_2_CO_3_ stress, while under NaHCO_3_ stress, the GR activity initially increased before gradually declining. Furthermore, under equal sodium ion conditions, the inhibition of the GR activity in the seedling roots was more pronounced with the Na_2_CO_3_ treatment than with the NaHCO_3_ treatment (Figure 9f). These results indicate that Na_2_CO_3_ stress has a considerably greater impact on plant hormone (auxin) signaling, ascorbic acid and uronate metabolism, glutathione metabolism, flavonoid biosynthesis, and other related pathways than the NaHCO_3_ treatment.

#### 3.6.2. Effects of Different Carbonate Stresses on the Expression of Genes Related to Auxin, Ascorbate, and Flavonoid Biosynthesis and Glutathione Metabolism in Rice Seedling Roots

**Figure 10:**
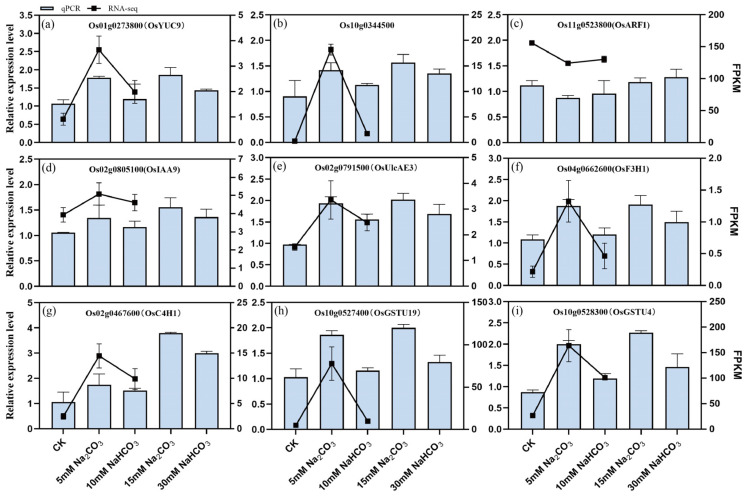
Validation of the expression pattern of the RNA-seq results by qRT-PCR in the roots of rice seedlings under different carbonate stresses: (**a**–**i**) relative expression levels of 9 genes, where the horizontal coordinate is the corresponding gene. The scale on the left axis represents the relative expression, and the right axis represents the FPKM value. The bars represent the results of the RT-qPCR, and the lines represent the results of the RNA-Seq. The data are the mean ± SD of three biological replicates.

We selected nine differentially expressed genes (DEGs) involved in plant hormone (auxin) signaling, ascorbate and uronate metabolism, glutathione metabolism, and flavonoid biosynthesis to validate the accuracy of our transcriptomic sequencing data using quantitative real-time PCR (qRT-PCR). The verification results are presented in Figure 10. Under Na_2_CO_3_ stress, we observed a significant upregulation of tryptophan metabolism genes (*Os01g0273800* and *Os10g0344500*) and the IAA response gene (*Os02g0805100*, *OsIAA9*). Additionally, ascorbic acid metabolism gene (*Os02g0791500*, *OsUlcAE3*), flavonoid biosynthesis genes (*Os04g0662600*, O*sF3H1*; *Os02g0467600*, *OsC4H1*), and glutathione metabolism-related genes (*Os10g0527400*, *OsGSTU19*; *Os10g0528300*, *OsGSTU4*) were significantly upregulated. In contrast, the IAA response gene (*Os11g0523800*, *OsARF1*) was downregulated. Notably, the expression levels of these genes did not show significant changes under the NaHCO_3_ treatment. These findings indicate that the gene expression patterns observed in the qRT-PCR align well with those obtained from the RNA-Seq data, confirming the reliability of our transcriptomic analysis.

## 4. Discussion

Soda-saline soil contains Na_2_CO_3_ and NaHCO_3_, which results in elevated pH levels. This increased pH is considered one of the primary factors contributing to alkali stress, which tends to cause more damage to plants than neutral salt stress [13,16,18]. To better understand the effects of different carbonates on plants, we used rice, a crop suitable for saline-alkali land, as the plant material to investigate the impacts of various carbonate stresses on the physiological, biochemical, and molecular characteristics of seedling roots.

Salinity-alkalinity stress imposes significant physiological constraints on plants, consequently limiting growth and agricultural productivity [41,42]. The impact of alkali stress on rice varies with the intensity of the stress. As the treatment concentration increases, rice seed germination and seedling growth are progressively inhibited, and seedlings damage becomes more severe [13]. Moreover, the responses among different rice varieties differ significantly. The alkali-tolerant variety (Dongdao 4) demonstrates higher seedling survival rates, greater plant height, and higher chlorophyll content compared to the alkali-sensitive variety (Jiudao 51) under alkali conditions. Additionally, Dongdao 4 experiences less damage and maintains lower levels of reactive oxygen species (ROS) while exhibiting higher antioxidant enzyme activities, indicating a stronger ability to withstand alkali stress [6]. This indicates the complexity of the differential effects of alkali stress on rice. The study investigated the effects of different carbonate stresses on rice seedling growth and root damage. Consistent with previous findings [43], both alkaline stresses suppressed rice seedling growth. The results also indicate that the Na_2_CO_3_ stress exhibited a markedly stronger inhibitory effect on rice seedlings compared with the NaHCO_3_ treatment (Figure 1), thus providing a foundation for understanding the differential physiological impacts of various carbonate types on rice seedlings. However, the degree of inhibition varied between the two carbonates, specifically, the seedling biomass in the high-concentration Na_2_CO_3_ treatment was significantly lower than those in both the control and NaHCO_3_ treatment groups (Figure 1c–f). This inhibitory effect is potentially linked to the severity of root cell membrane damage, as evidenced by increased electrolyte leakage, indicative of compromised membrane integrity (Figure 1g). Damage to the plasma membrane may affect the function of membrane proteins and cell signaling processes [44], thereby interfere with growth regulation. Furthermore, compared to the NaHCO_3_ treatment, the Na_2_CO_3_ stress led to a significant reduction in root vigor (Figure 1h), further confirming that Na_2_CO_3_ stress has a more detrimental effect on rice roots.

Oxidative stress, a significant contributor to plant damage under abiotic stress, is often analyzed as a key physiological and biochemical indicator [45,46]. For instance, drought, salt, and alkaline stresses induce reactive oxygen species (ROS) accumulation in many plants [47,48,49]. In this study, both ROS and malondialdehyde (MDA) levels significantly increased in rice roots under different carbonate stresses, indicating that carbonate stress induced oxidative stress. Particularly, ROS and MDA levels were markedly higher with the Na_2_CO_3_ treatment compared to the NaHCO_3_ treatment. The excessive production of ROS and MDA likely contributed to root cell membrane damage (Figure 1g) and reduced root vigor (Figure 1h). Concurrently, different carbonate stresses increased the activity of antioxidant enzymes, such as superoxide dismutase (SOD), peroxidase (POD), and catalase (CAT). While changes in these enzyme activities were minimal in the low-concentration carbonate treatments, they exhibited a significant decrease in the high-concentration treatments, especially in the Na_2_CO_3_ treatment. These results further demonstrate that the high-concentration Na_2_CO_3_ treatments suppressed the antioxidase system of the rice seedlings under intense oxidative pressure, which contributed to the accumulation of ROS and high MDA content in the roots (Figure 2a–c). This also indicates that Na_2_CO_3_ caused more severe oxidative stress damage to rice roots.

Omics analysis is a common and effective means to analyze the differences among a wide range of treatments [50]. By transcriptome analysis, we identified significant changes in gene expression in rice roots subjected to carbonate stress. The results of the GO and KEGG enrichment analyses indicated that differentially expressed genes in the Na_2_CO_3_ treatment group were primarily associated with biological processes related to responses to stimuli and chemicals. In contrast, the NaHCO_3_ treatment group featured genes that were predominantly linked to components associated with cell membranes. This suggests that there are distinct differences in the response mechanisms of rice roots to varying carbonate stresses. Additional, non-targeted metabolome analysis revealed a significant impact of alkali stress on the metabolite composition of rice roots. Our findings indicate that the changes in metabolites induced by carbonate stress are closely related to the growth and adaptation mechanisms of the plant. In particular, in the s Na_2_CO_3_ treatment group, the enriched metabolite pathways were associated with amino acid metabolism, carbon metabolism, and unsaturated fatty acid biosynthesis. Similar to observations by Wang et al. and Zhou et al. [51,52], the primary pathways enriched in differentially abundant metabolites were amino acid metabolism and the TCA cycle. These pathways may play a crucial role in the ability of rice to adapt to saline-alkali stress.

Joint analysis of the transcriptome and metabolome data revealed common enriched pathways across the two comparison groups, including the metabolism of alanine, aspartate, glutamate, carbon, and glyoxylate. These enriched pathways are consistent with omics analyses of alkali stress in other studies [7,53]. Differentially expressed genes (DEGs) and differentially accumulated metabolites (DEMs) were notably enriched in pathways related to plant hormone (auxin) signaling, ascorbic acid and uronate metabolism, glutathione metabolism, and flavonoid biosynthesis, specifically in the comparison of the CK versus Na treatment groups (Figure 2 and Figure 8), highlighting their close association with plant growth and reactive oxygen species (ROS) clearance. This study investigated the effects of Na_2_CO_3_ and NaHCO_3_ on the synthesis of plant hormones and antioxidant substances, as well as the expression of related genes, by validating both metabolite profiles and gene expression in the roots of rice seedlings under varying carbonate stress. Our findings indicate that as the duration of Na_2_CO_3_ stress increased the content of indole-3-acetic acid (IAA) in the roots of rice seedlings progressively decreased. The inhibitory effect on IAA was significantly stronger with the high-concentration Na_2_CO_3_ treatment group compared to the NaHCO_3_ group. Tryptophan serves as a precursor for the biosynthesis of the plant hormone IAA, and the regulation of tryptophan metabolic pathways may affect auxin synthesis [54]. At the transcriptional level, genes involved in tryptophan metabolism (*Os01g0273800* and *Os10g0344500*) and the IAA-responsive gene *OsIAA9* were significantly upregulated under Na_2_CO_3_ stress (Figure 10a,b,d), whereas the IAA response gene *OsARF1* was downregulated (Figure 10c). These expression patterns corroborated the reliability of the transcriptomic analysis validated by qRT-PCR. Consistent with our findings, previous studies have demonstrated that alkali stress affects the expression of IAA biosynthesis and signaling genes and elevates endogenous IAA levels [43,55]. Collectively, these results suggest that Na_2_CO_3_ stress may influence rice growth and development by disrupting IAA synthesis and the expression of its response genes.

Our results also reveal that alkaline stress impacts the synthesis of non-enzymatic antioxidant compounds in rice seedling roots, providing valuable insight into the physiological responses of rice under alkaline stress. Marked increases in the accumulation of H_2_O_2_ and O_2_^−^ were observed after alkaline stress treatment 1 d (Figure 2a,b). Concomitantly, the contents of ascorbic acid and flavonoids in the rice roots treated with Na_2_CO_3_, along with the expression levels of genes related to their metabolism, were also significantly elevated (Figure 10e–g). This likely represents a self-protective mechanism, whereby plants initiate antioxidant synthesis in response to oxidative stress [23]. While flavonoid content increased significantly after Na_2_CO_3_ treatment 1 d, accompanied by a corresponding increase in the expression of flavonoid biosynthesis genes *Os04g0662600* (*OsF3H1*) and *Os02g0467600* (*OsC4H1*). These expression patterns and contents certified the accuracy of the transcriptomic and metabolome results again. Subsequently, the flavonoid levels gradually decreased with prolonged treatment. This phenomenon may be related to changes in the antioxidant requirements of plants. Similar results have been reported in sorghum [56], suggesting that rice faces challenges in maintaining its antioxidant capacity under Na_2_CO_3_ stress. In addition, a significant increase in glutathione levels (GSH and GSSG) was observed under alkaline stress. With the increasing stress duration, the glutathione content gradually increased, and accordingly, the expression levels of glutathione S-transferase genes *Os10g0527400* (*OsGSTU19*) and *Os10g0528300* (*OsGSTU4*) were upregulated. Consistent with these findings, overexpression of *OsGSTU4* in *Arabidopsis* has been shown to enhance tolerance to salt and oxidative stress [57]. These results suggest that rice seedlings may employ enhanced glutathione synthesis as a mechanism to protect cells against oxidative stress. However, the activity of glutathione reductase (GR) was significantly reduced in the Na_2_CO_3_-treated group, suggesting that Na_2_CO_3_ stress may lead to dysregulation of glutathione metabolism, thereby affecting its antioxidant function in rice seedling roots.

## 5. Conclusions

In summary, the results of this study indicate that Na_2_CO_3_ stress, representing high pH, has a more significant impact on the growth and physiological characteristics of rice seedlings, particularly regarding plant hormone synthesis and antioxidant metabolic pathways compared with NaHCO_3_. These findings offer a fresh perspective for further investigations into the mechanisms by which rice adapts to an alkali environment, providing an important theoretical foundation for future breeding and agricultural management strategies. Future research should delve deeper into the molecular mechanisms underlying the enhancement of antioxidant capacity in rice under alkaline stress. This will contribute to mitigating oxidative damage to rice in actual saline-alkali field production, as well as support efforts to improve the alkali resistance of rice.

## Data Availability

Data are contained within the article and Supplementary Materials.

## Supplementary Materials

The following supporting information can be downloaded at: https://www.mdpi.com/article/10.3390/antiox14060658/s1, Table S1: Evaluation statistics of the sequencing data of the underground samples of *Oryza sativa* L.; Table S2: The top ten GO pathways of the DEGs by enrichment degree; Table S3: The top ten KEGG pathways of the DEGs by enrichment degree; Table S4; The top ten KEGG pathways of the DAMs by enrichment degree; Table S5: The top ten KEGG pathways of the DEGs and DAMs by enrichment degree; Table S6: The four KEGG pathways of the DEGs and DAMs by enrichment degree; Table S7: Nucleotide sequences of the RT-qPCR primers.
