# Supplementary material for: Integrated Transcriptome and Metabolome Analyses Reveal Complex Oxidative Damage Mechanisms in Rice Seedling Roots Under Different Carbonate Stresses
Source: Antioxidants (Basel). 2025 May 30;14(6):658. doi: 10.3390/antiox14060658 (PMC12190083; doi:10.3390/antiox14060658)
Supplement: Supplementary file 1 [file antioxidants-14-00658-s001.zip › antioxidants-3619104-supplementary.pdf]

Supplementary Materials

Table S1. Evaluation statistics of sequencing data of underground samples of *Oryza sativa L.*

Sample	Raw_reads	Raw_bases	Clean_reads	Clean_bases	Q20	Q30	GC content
CK1	55823074	8.4G	55484614	8.38G	98.07%	94.37%	52.65%
CK2	48448024	7.26G	47974942	7.24G	97.94%	94.04%	52.08%
CK3	50467992	7.58G	50119914	7.57G	97.84%	93.81%	52.69%
Na1	54994352	8.26G	54589948	8.24G	98.15%	94.66%	52.77%
Na2	53365180	7.98G	52769944	7.97G	98.15%	94.59%	53.32%
Na3	51477316	7.73G	51097290	7.72G	98.00%	94.27%	52.72%
NaH1	53124764	7.99G	52796684	7.97G	97.98%	94.16%	53.14%
NaH2	50201746	7.54G	49826208	7.52G	98.11%	94.55%	53.21%
NaH3	50587120	7.55G	49909662	7.54G	97.92%	93.95%	52.00%

Table S2. The top ten GO pathways of DEGs enrichment degree.

CK vs Na

Ontology	ID	Description	GeneRatio	pvalue	Count
Cellular Component	GO:0005576	extracellular region	98/2160	7.00E-19	98
Molecular Function	GO:0004568	chitinase activity	19/2160	2.01E-13	19
Biological Process	GO:0016998	cell wall macromolecule catabolic process	18/2160	8.81E-12	18
Biological Process	GO:0050896	response to stimulus	272/2160	6.13E-11	272
Biological Process	GO:0042221	response to chemical	122/2160	1.37E-09	122
Molecular Function	GO:0016491	oxidoreductase activity	148/2160	1.80E-09	148
Molecular Function	GO:0003824	catalytic activity	575/2160	5.81E-08	575
Cellular Component	GO:0009538	photosystem I reaction center	5/2160	2.34E-07	5
Biological Process	GO:0044347	cell wall polysaccharide catabolic process	11/2160	4.03E-07	11
Molecular Function	GO:0046906	tetrapyrrole binding	57/2160	4.37E-07	57

CK vs NaH

Ontology	ID	Description	GeneRatio	pvalue	Count
Cellular Component	GO:0005576	extracellular region	62/1218	1.05E-14	62
Molecular Function	GO:0004568	chitinase activity	16/1218	1.39E-14	16
Cellular Component	GO:0031224	intrinsic component of membrane	241/1218	6.62E-14	241
Cellular Component	GO:0016021	integral component of membrane	232/1218	3.34E-12	232
Cellular Component	GO:0016020	membrane	284/1218	1.61E-10	284
Biological Process	GO:0050896	response to stimulus	159/1218	3.89E-10	159
Biological Process	GO:0042221	response to chemical	74/1218	1.20E-08	74
Biological Process	GO:0016998	cell wall macromolecule catabolic process	11/1218	4.74E-08	11
Molecular Function	GO:0046906	tetrapyrrole binding	37/1218	2.09E-07	37
Molecular Function	GO:0020037	heme binding	36/1218	2.49E-07	36

Table S3. The top ten KEGG pathways of DEGs enrichment degree.

CK vs Na

Category	ID	Description	GeneRatio	pvalue	Count
KEGG	ko01100	Metabolic pathways	178∣273	3.76E-09	178
KEGG	ko01110	Biosynthesis of secondary metabolites	110∣273	5.12E-06	110
KEGG	ko00520	Amino sugar and nucleotide sugar metabolism	23∣273	8.31E-06	23
KEGG	ko00940	Phenylpropanoid biosynthesis	25∣273	1.82E-05	25
KEGG	ko00195	Photosynthesis	12∣273	5.01E-04	12
KEGG	ko00430	Taurine and hypotaurine metabolism	5∣273	9.66E-04	5
KEGG	ko00960	Tropane, piperidine and pyridine alkaloid biosynthesis	7∣273	0.001408172	7
KEGG	ko04016	MAPK signaling pathway - plant	18∣273	0.007153786	18
KEGG	ko00350	Tyrosine metabolism	7∣273	0.01054609	7
KEGG	ko00950	Isoquinoline alkaloid biosynthesis	5∣273	0.01154053	5

CK vs NaH

Category	ID	Description	GeneRatio	pvalue	Count
KEGG	ko00940	Phenylpropanoid biosynthesis	19∣119	4.66E-08	19
KEGG	ko00520	Amino sugar and nucleotide sugar metabolism	17∣119	9.58E-08	17
KEGG	ko04016	MAPK signaling pathway - plant	13∣119	2.49E-04	13
KEGG	ko01110	Biosynthesis of secondary metabolites	51∣119	3.25E-04	51
KEGG	ko00430	Taurine and hypotaurine metabolism	4∣119	3.58E-04	4
KEGG	ko00904	Diterpenoid biosynthesis	6∣119	7.81E-04	6
KEGG	ko01100	Metabolic pathways	74∣119	1.21E-03	74
KEGG	ko00965	Betalain biosynthesis	2∣119	6.23E-03	2
KEGG	ko00350	Tyrosine metabolism	4∣119	2.17E-02	4
KEGG	ko00950	Isoquinoline alkaloid biosynthesis	3∣119	2.23E-02	3

Table S4. The top ten KEGG pathways of DAMs enrichment degree.

CK vs Na

Category	ID	Description	GeneRatio	pvalue	Count
KEGG	ko00250	Alanine, aspartate and glutamate metabolism	4∣230	5.24E-03	4
KEGG	ko01200	Carbon metabolism	4∣230	5.24E-03	4
KEGG	ko00020	Citrate cycle (TCA cycle)	3∣230	1.98E-02	3
KEGG	ko00630	Glyoxylate and dicarboxylate metabolism	3∣230	1.98E-02	3
KEGG	ko00710	Carbon fixation in photosynthetic organisms	3∣230	1.98E-02	3
KEGG	ko00760	Nicotinate and nicotinamide metabolism	3∣230	1.98E-02	3
KEGG	ko00660	C5-Branched dibasic acid metabolism	2∣230	0.07415986	2
KEGG	ko00591	Linoleic acid metabolism	4∣230	0.09145586	4
KEGG	ko00220	Arginine biosynthesis	2∣230	0.1827976	2
KEGG	ko00261	Monobactam biosynthesis	2∣230	0.1827976	2

CK vs NaH

Category	ID	Description	GeneRatio	pvalue	Count
KEGG	ko00710	Carbon fixation in photosynthetic organisms	3∣230	1.30E-03	3
KEGG	ko01200	Carbon metabolism	3∣230	4.80E-03	3
KEGG	ko01040	Biosynthesis of unsaturated fatty acids	4∣230	1.11E-02	4
KEGG	ko00660	C5-Branched dibasic acid metabolism	2∣230	1.23E-02	2
KEGG	ko00020	Citrate cycle (TCA cycle)	2∣230	3.44E-02	2
KEGG	ko00261	Monobactam biosynthesis	2∣230	3.44E-02	2

<table>
<tr><td>KEGG</td><td>ko00620</td><td>Pyruvate metabolism</td><td>2|230</td><td>3.44E-02</td><td>2</td></tr>
<tr><td>KEGG</td><td>ko00630</td><td>Glyoxylate and dicarboxylate metabolism</td><td>2|230</td><td>3.44E-02</td><td>2</td></tr>
<tr><td>KEGG</td><td>ko00760</td><td>Nicotinate and nicotinamide metabolism</td><td>2|230</td><td>3.44E-02</td><td>2</td></tr>
<tr><td>KEGG</td><td>ko00250</td><td>Alanine, aspartate and glutamate metabolism</td><td>2|230</td><td>6.40E-02</td><td>2</td></tr>
</table>

Table S5. The top ten KEGG pathways of DEGs and DAMs enrichment degree.

CK vs Na

Category	ID	Description	P-value_gene	P-value_meta
KEGG	ko00250	Alanine, aspartate and glutamate metabolism	0.06343695	5.24E-03
KEGG	ko01200	Carbon metabolism	0.4715556	5.24E-03
KEGG	ko00630	Glyoxylate and dicarboxylate metabolism	0.7988415	1.98E-02
KEGG	ko00710	Carbon fixation in photosynthetic organisms	0.06420839	1.98E-02
KEGG	ko00760	Nicotinate and nicotinamide metabolism	0.6955827	1.98E-02
KEGG	ko00660	C5-Branched dibasic acid metabolism	0.3903896	7.42E-02
KEGG	ko00220	Arginine biosynthesis	0.2636996	0.1827976
KEGG	ko00620	Pyruvate metabolism	0.7852995	0.1827976
KEGG	ko00906	Carotenoid biosynthesis	0.02495986	0.206584
KEGG	ko01040	Biosynthesis of unsaturated fatty acids	0.3306005	0.2093467

CK vs NaH

Category	ID	Description	GeneRatio	pvalue
KEGG	ko01110	Biosynthesis of secondary metabolites	3.25E-04	0.9643193
KEGG	ko00430	Taurine and hypotaurine metabolism	3.58E-04	0.1130435
KEGG	ko01100	Metabolic pathways	1.21E-03	0.7980848
KEGG	ko00350	Tyrosine metabolism	2.17E-02	0.09935196
KEGG	ko00360	Phenylalanine metabolism	2.78E-02	0.3831926
KEGG	ko00410	beta-Alanine metabolism	8.32E-02	0.3831926
KEGG	ko00250	Alanine, aspartate and glutamate metabolism	1.27E-01	0.0640484
KEGG	ko00270	Cysteine and methionine metabolism	1.41E-01	0.06810512
KEGG	ko00460	Cyanoamino acid metabolism	1.41E-01	0.3831926
KEGG	ko00052	Galactose metabolism	1.51E-01	0.4541527

Table S6. The four KEGG pathways of DEGs and DAMs enrichment degree.

CK vs Na

Category	ID	Description	P-value_gene	P-value_meta
KEGG	ko04075	Plant hormone signal transduction	0.1378942	2.74E-01
KEGG	ko00053	Ascorbate and aldarate metabolism	0.9735853	4.74E-01
KEGG	ko00480	Glutathione metabolism	0.0223708	4.74E-01
KEGG	ko00941	Flavonoid biosynthesis	0.3574368	8.02E-01

Table S7. Nucleotide sequences of RT-qPCR primers.

Gene name	Locus	Primer sequences(5'→3')
OsYUC9	*Os01g0273800*	Forward:GGATGGTGAGGAGGGGAA
		Reverse:CATTGTTGTGGGTAGGCTT
	Os10g0344500	Forward:GTGGTCGTCGTTTGAGATAC
		Reverse:CTGATTGACAGAACAGCAGTT
OsARF1	*Os11g0523800*	Forward:GATGGTGCTTGTCCTTGGTG
		Reverse:CGGCTGACAACTTACCTCAC
OsIAA9	*Os02g0805100*	Forward:GAAGGAGATGCATGCGTGAG

		Reverse:CATACATCAGTGCACACCCA
OsUGlcAE3	*Os02g0791500*	Forward:GAAGGTGAAGATCGAGAGGG<br>Reverse:CGACGAAGGACTGGAAGC
OsF3H1	*Os04g0662600*	Forward:GCGTGCGAGGAGTGGGG<br>Reverse:TGGCTGGAGACGATGAA
OsC4H1	*Os02g0467600*	Forward:CTTCTTGCGGGGCTACCT<br>Reverse:GTCCATCACCTTCCTTCTCT
OsGSTU19	*Os10g0527400*	Forward:CGTCACCCTCCTCCCCG<br>Reverse:ACCGCCTCCGCCTTCTC
OsGSTU4	*Os10g0528300*	Forward:CCGTCGCTCGCTTCTGG<br>Reverse:TCCCCTCCGCCTTCTCC
Osβ-actin	*Os03g0718100*	Forward:TTCCAGCCTTCCTTCATA<br>Reverse:AACGATGTTGCCATATAGAT
